# The Limit Tuning Effects Exerted by the Mechanically Induced Artificial Potential Barriers on the *I*–*V* Characteristics of Piezoelectric PN Junctions

**DOI:** 10.3390/mi13122103

**Published:** 2022-11-29

**Authors:** Yizhan Yang, Jiankang Chen, Yunbo Wang

**Affiliations:** 1 Department of Mechanics, School of Aerospace Engineering, Hubei Key Laboratory of Engineering Structural Analysis and Safety Assessment, Huazhong University of Science and Technology, Wuhan 430074, China; 2 School of Mechanical Engineering and Mechanics, Zhejiang Provincial Engineering Research Center for the Safety of Pressure Vessel and Pipeline, Ningbo University, Ningbo 315211, China; 3 Department of Microelectronics, School of Optical and Electronic Information, Huazhong University of Science and Technology, Wuhan 430074, China

**Keywords:** piezoelectric PN junction, artificial potential barrier, limit tuning effect, multiple competition

## Abstract

A mechanically induced artificial potential barrier (MIAPB) in piezoelectric semiconductor devices is set up under the action of a pair of tensile/compressive mechanical loadings. Three factors, namely, the barrier height, width and position, affect the nature and extent of interaction between the MIAPB and the contact barrier, and the tuning characteristics (generated under conditions of the artificial barrier) of the piezoelectric PN junctions were studied. The influence of these factors resulted in variations in the interaction intensities, superposition effects, carrier inversion degrees and carrier redistribution ranges. Subsequently, the limit tuning effects exerted by the tensile/compressive-mode MIAPB on the PN junctions were studied. The inconsistency between the left and right end of the tensile-mode MIAPB under conditions of the offset loading state proves that the maximum tuning effect is generated when both sides of the interface are symmetrically loaded. The range of carrier redistribution and the over-inversion of local carriers, affected by the width and height of MIAPB, result in a second competitive mechanism. The carrier redistribution range and the carrier inversion degree require that the compressive-mode MIAPB be sufficiently wide. The interaction intensities and the superposition effects, affected by the position and height of the MIAPB, contribute to the second competing mechanism. We logically clarify the relationship between multiple competition and find that the emergence of multiple competitive mechanisms proves the existence of the limit tuning effect of MIAPB on the *I*–*V* properties of PN junctions. The results reported herein provide a platform for understanding the mechanical tuning laws governing the functions of piezoelectric PN junctions and piezoelectric devices.

## 1. Introduction

Researchers have widely investigated the coupling effects generated by the piezoelectric and semiconductor effects. The results have helped design and fabricate smart materials and functional material-based devices. Nanogenerators [1,2], piezoelectric transducers [3], piezoelectric semiconductor sensors [4,5,6], acoustic wave amplifiers [7,8,9] and acoustic-electric conversion devices [10,11,12] are typical examples of devices that have been developed over the years. Currently, flexible electronic devices and third-generation semiconductors are extensively researched to develop self-powered and wearable piezotronic devices. This will eventually result in the development of the field associated with the fabrication of semiconductor devices [13]. Piezoelectric semiconductors are often used as energy and signal conversion units as they exhibit the electromechanical coupling effect. Since Wang et al. [14,15] proposed the concept of “piezotronics”, research on piezoelectric semiconductors has entered a new stage. In-depth research is being conducted on the piezoelectric semiconductors, and the mechanical [16], thermal [17,18], magnetic [19] and optical [20] loading of the systems are being extensively studied. The responses of the devices have also attracted immense attention. The development of piezoelectric devices can potentially promote the development of micro-nano sensors, MEMS, nano-robots and flexible electronic devices. It can also improve the extent of human–computer interaction realized [13,21].

Analysis of the piezoelectric effect reveals that piezoelectric semiconductor devices are significantly affected by mechanical loadings, such as bending, torsion, tension, compression and vibration, when these are used or transported [22,23,24,25]. In addition, the external loadings can also induce the coupling between the mechanical response and the charge carriers, such as the formation of carrier-coupled waves [26], or the wave-particle drag effect [27]. Hence, it is important to study the response generated by piezoelectric semiconductor devices under the action of mechanical loadings and the influence of the loadings on related electrical quantities to understand the working properties of functional devices fabricated using piezoelectric semiconductors. Huang et al. [28] studied the effect exerted by mechanical loadings on the *I*–*V* curves generated for piezoelectric semiconductor fibers. Under the action of the polarized electric field, the local tensile/compressive loadings associated with the fiber create a local artificial barrier, which can potentially prevent the passage of the charge carriers through the stressed region. The results obtained from the reference [28] are consistent with the results reported by Fan et al. [29]. They worked on the linear analysis of electrical potential and carrier distribution induced by the action of mechanical loadings.

The PN junction is the core component of modern electronic devices. The depletion layer approximation method [30,31] is used to simplify the calculation when the classical PN junction theoretical model is considered. However, numerous intrinsic and interesting properties associated with the contact barrier region are ignored during the calculations [32]. Yang et al. [32,33] established a fully coupled model based on piezoelectric constitutive equations, Gauss’s law and current continuity equations and opened the space charge region. Then they proposed a mechanical tuning technique for barrier configuration. In many studies on non-thermal equilibrium piezoelectric semiconductor devices, the recombination of the carriers in the MIAPB region is often ignored to simplify the calculation method [28,34]. This helps in the study of the individual n-type doped or p-type doped piezoelectric semiconductors, which can be attributed to the fact that the concentration of minority carriers is significantly low under these conditions. But this simplification is not feasible for electronic devices with a large number of non-thermal equilibrium minority carriers, such as PN junctions. And the applied mechanical loading will inevitably affect the distribution of non-equilibrium carriers within the device when considering the piezoelectric effect, thereby affecting its recombination behavior and electrical properties. Yang et al. [33] revealed the relationship between recombination rate and mechanical tuning of the *I*–*V* characteristics of the PN junction. On this basis, Yang et al. [35] studied the detailed mechanism of the tension- and compression-mode MIAPB tuning effect on the PN junction. However, the factors affecting the regulatory effect of MIAPB and the conditions under which there is the limit tuning effect of MIAPB on the PN junction have not been fully elucidated.

Herein, the three main factors (barrier height, width and position) that affect the tuning effects generated by mechanically induced artificial barriers on the *I*–*V* characteristics of piezoelectric PN junctions are reported. It has been observed that the three factors are coupled to each other, resulting in the generation of different interaction intensities, superposition effects, carrier inversion degrees and carrier redistribution ranges. Thus, different modes of MIAPB result in the development of diverse and multiple competitive phenomena that result in current enhancement and weakening. This forms the physical basis of the limit tuning effect. Finally, the most significant effects exerted by MIAPB on the *I*–*V* characteristics of piezoelectric PN junctions under the compressive and tensile modes are analyzed, and the mechanism is explained from the viewpoint of optimized carrier recombination.

## 2. Equations Governing a Piezoelectric PN Junction

**Figure 1:**
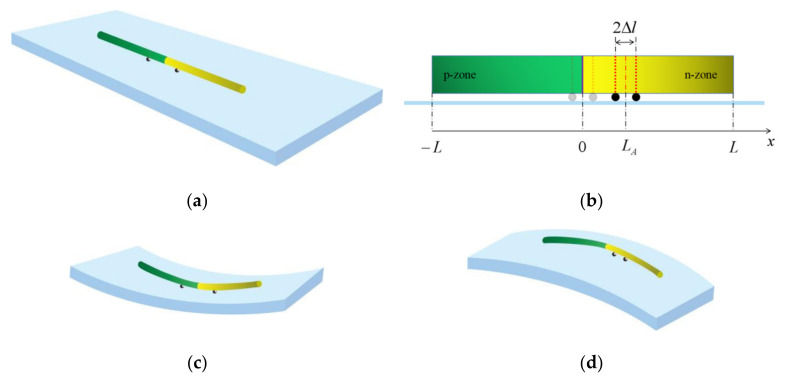
MIAPB formed by a pair of tensile/compressive stresses for the case of a PN junction in a ZnO system. (**a**) Unloaded 3D PN junction structure, (**b**) Front view of the structure, (**c**) Compressive-mode MIAPB induced by bending substrate up and (**d**) Tensile-mode MIAPB induced by bending substrate down.

Figure 1 presents the schematic representation of a piezoelectric PN junction consisting of two p- and n-type doped ZnO rods (fixed interface at x=0) and the method to apply mechanical loadings. Figure 1a shows the three-dimensional structure, and the PN junction is connected to the elastic substrate through solder joints. In Figure 1b, the one-dimensional Cartesian coordinate system is established, and relevant geometric parameters are marked. Different modes of MIAPB need to act on different loading positions to achieve better regulatory effects. Therefore, MIAPB needs to move within a certain range of the PN junction to obtain the best tuning effect [33,35]. The gray points represent the initial loading positions, and the black dots represent the final loading positions. The length of the PN junction is denoted by 2L, and the polarization direction runs from left to right. The distance between the center of the MIAPB and the origin is denoted by $L_{A}$ when MIAPB is symmetrically loaded on both sides of the interface ($L_{A}=0$). The range of mechanical loading that generates the MIAPB is 2Δl. Figure 1c,d graphically illustrates the way of loading, and compressive/tensile loading is achieved by bending up or down [14]. The axial size of the PN junction is significantly larger than that associated with the lateral dimension. This can be simplified as a one-dimensional structure for efficient analysis.

The electrostatic Gauss’s law is represented as follows [33]:

(1) $$\frac{dD}{dx}=\left\{\begin{array}{l} q(\Delta p_{p}(x)-\Delta n_{p}(x)),\;x\in [-L,0],\\ q(\Delta p_{n}(x)-\Delta n_{n}(x)),\;x\in [0,L], \end{array}\right.$$

where D denotes the electrical displacement; $q(1\times 10^{-19}C)$ is the electron charge; and Δp and Δn represent the increments in the number of holes and electrons, respectively. The subscripts “*p*” and “*n*” represent the p-zone and the n-zone, respectively. When a tensile/compressive loading is applied at a partial PN junction, D is expressed as follows:

(2) $$D=\left\{\begin{array}{l} (\hat{e}/\hat{c})\sigma +\bar{\varepsilon }E,\;x\in (L_{A}-\Delta l,L_{A}+\Delta l),\\ \bar{\varepsilon }E,\;x\notin (L_{A}-\Delta l,L_{A}+\Delta l), \end{array}\right.$$

where E is the electric field and $\bar{\varepsilon }=(1+\xi^{2})\hat{\varepsilon }$, $\xi^{2}={\hat{e}}^{2}/(\hat{c}\hat{\varepsilon })$. Only three normal strains are considered for the case of a one-dimensional PN junction model, and the shear strain can be ignored under this condition. Therefore, the stress can be released (as described in literature reports) [27]. The constitutive model is expressed as follows:

(3) $$\begin{array}{l} \sigma =\hat{c}S-\hat{e}E,\\ D=\hat{e}S+\bar{\varepsilon }E, \end{array}$$

where $\hat{c}=c_{33}-2c_{13}^{2}/(c_{11}+c_{12})$, $\hat{e}=e_{33}-2e_{31}c_{13}/(c_{11}+c_{12})$ and $\hat{\varepsilon }=\varepsilon_{33}+2e_{31}^{2}/(c_{11}+c_{12})$. The symbols $c_{11}$, $c_{12}$, $c_{13}$ and $c_{33}$ represent the elastic moduli; $e_{31}$ and $e_{33}$ are piezoelectric constants; and $\varepsilon_{33}$ is the dielectric constant.

The current density described by the classical drift-diffusion model is expressed as follows:

(4) $$j_{p}=qp\mu_{p}E-qD_{p}\frac{dp}{dx},\;j_{n}=qn\mu_{n}E+qD_{n}\frac{dn}{dx},$$

where $\left(\mu_{p},D_{p}\right)$ and $\left(\mu_{n},D_{n}\right)$ represent the mobility and diffusion coefficient of the holes and electrons, respectively. These satisfy the Einstein relationship $D_{\left(n,p\right)}=\mu_{\left(n,p\right)}kT/q$. The current continuity conditions associated with the PN junction are considered, and the interface and the loading positions are taken into account. The equations obtained under these conditions are as follows:

(5) $$\begin{array}{l} -\mu_{n}n\frac{d^{2}\phi }{dx^{2}}-\mu_{n}\frac{dn}{dx}\frac{d\phi }{dx}+D_{n}\frac{d^{2}n}{dx^{2}}-R=0,\\ \mu_{p}p\frac{d^{2}\phi }{dx^{2}}+\mu_{p}\frac{dp}{dx}\frac{d\phi }{dx}+D_{p}\frac{d^{2}p}{dx^{2}}-R=0. \end{array}$$

When analyzing a semiconductor device with a large number of minority-carrier injections like a PN junction, the recombination serves as the main mechanism for the conversion of the majority and minority carrier current; its intensity can significantly affect the magnitude of the entire current density. The Shockley–Read–Hall recombination model is used for analysis, and it is assumed that the recombination center is in the middle of the forbidden band. Under these conditions, the recombination rate is obtained as follows:

(6) $$R=\frac{np-n_{i}^{2}}{\tau_{p}(n+n_{i})+\tau_{n}(p+n_{i})},$$

where $n_{i}$ is the intrinsic carrier concentration at the PN junction, and $\tau_{p}\;\mathrm{and}\;\tau_{n}$ represent the lifetimes of the holes and electrons, respectively.

Under conditions of complete ionization of impurities, the following relation is satisfied:

(7) $$(p_{p0},n_{n0})=(N_{A},N_{D}),$$

$V_{D}^{0}$ represents the contact potential difference corresponding to the initial contact potential barrier, and it can be expressed as follows:

(8) $$V_{D}^{0}=2.3(kT/q)\log(N_{D}N_{A}/n_{i}^{2}).$$

Our model does not consider the presence of a depletion layer, and the low injection conditions are overlooked. Under these conditions, the extent of the space charge region can be used as a reference size. This can be expressed as follows:

(9) $$l_{0}=\sqrt{2\bar{\varepsilon }V_{D}^{0}(N_{A}+N_{D})/(qN_{A}N_{D})}/2.$$

Yang et al. [33] found that, due to the shielding effect of carriers on polarized electric field (situated far away from the loading points), the concentration of carriers is comparable to the unloaded state. L is considered equal to $80l_{0}$(66.28 μm). The length of the model ensures that the two ends are under conditions of the thermal equilibrium states. The boundary conditions are as follows:

(10) $$\begin{array}{l} {\left.\phi \right|}_{\bar{x}=-80}=0,\;{\left.\Delta n_{p}\right|}_{\bar{x}=-80}=0,\;{\left.p_{p}\right|}_{\bar{x}=-80}=p_{p0},\\ {\left.\phi \right|}_{\bar{x}=80}=V_{D}^{0}-V,\;{\left.\Delta p_{n}\right|}_{\bar{x}=80}=0,\;{\left.n_{n}\right|}_{\bar{x}=80}=n_{n0}, \end{array}$$

where $\bar{x}=x/l_{0}$ and V is the external voltage (0.6 V). It is assumed that the two ends of the PN junction are in ohmic contact. The ZnO PN junction is taken as a representative example. The relevant material parameters are as follows [35,36,37]:

(11) $$\begin{array}{l} c_{11}=209.7\mathrm{GPa},\;c_{12}=121.1\mathrm{GPa},\;c_{11}=105.1\mathrm{GPa},\;c_{11}=210.9\mathrm{GPa}\\ e_{31}=0.537{C/m}^{2},\;e_{33}=-1.32{C/m}^{2},\;\varepsilon_{33}=9.16\varepsilon_{0}\\ \mu_{n}=0.0205m^{2}/\mathrm{Vs},\;\mu_{p}=0.007m^{2}/\mathrm{Vs}. \end{array}$$

The doping concentration is expressed as $N_{D}=N_{A}=1\times 10^{21}{\mathrm{cm}}^{-3}$, with $n_{i}=10\times 10^{12}{\mathrm{cm}}^{-3}$. The lifetime of holes and electrons is expressed as $\tau_{p}=\tau_{n}=1\;\mu s$.

## 3. Limit Tuning Effect Exerted by MIAPB on the *I*–*V* Characteristics of a PN Junction

### 3.1. Three Factors Affecting the Tuning Effect

When the polarization electric field drives the redistribution of carriers, the concentration difference between the majority and minority carriers changes with the loading position. This can be attributed to the uneven distribution of carrier charges in the PN junction. It can be inferred that, under conditions of carrier recombination, the position of MIAPB significantly influences the *I*–*V* characteristics of piezoelectric PN junctions. The width of MIAPB determines whether the internal carriers can completely shield the polarization electric field within it. It also affects the regulation effect exerted by MIAPB on the contact barrier structure. The application of different magnitudes of force results in the generation of different magnitudes of polarization electric fields in a piezoelectric PN junction. This results in the generation of MIAPBs with different barrier heights. Therefore, for consistency, the height of the barrier is described by stress and measured in MPa rather than V or eV. Thus, the barrier, position, width and height are the three factors that influence the tuning effect exerted on the *I*–*V* characteristics of piezoelectric PN junctions.

### 3.2. Limit Tuning Effect Exerted by Tensile-Mode MIAPB

**Figure 2:**
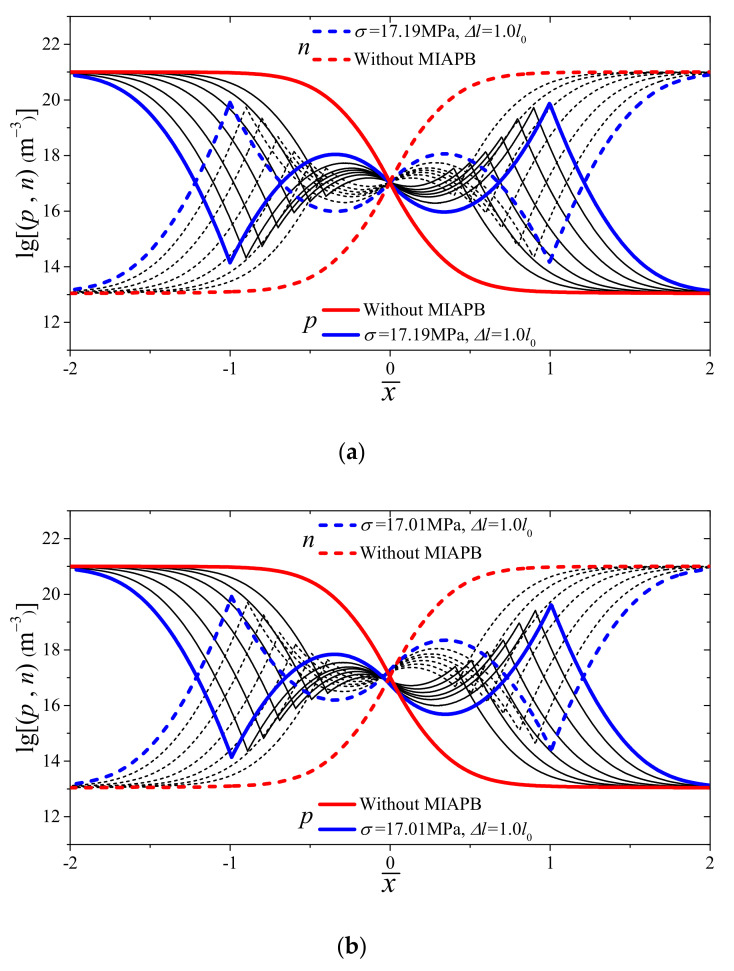

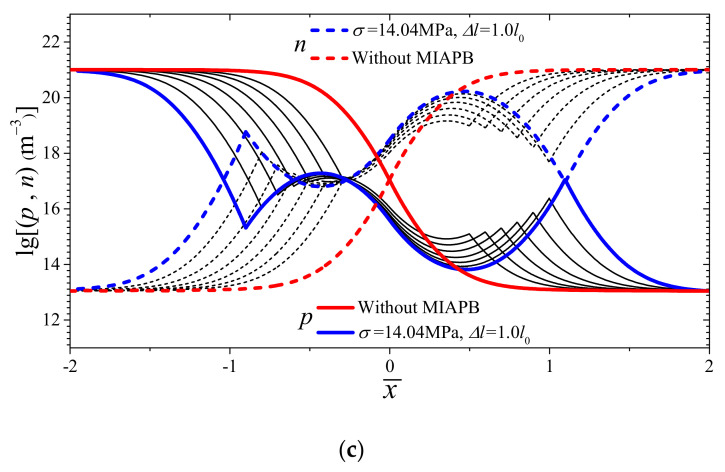
Evolutionary process of carrier concentration corresponding to the maximum output current under different offset conditions when Δl varies from $0.4l_{0}$ to $1.0l_{0}$. (**a**) $L_{A}/l_{0}=0$; (**b**) $L_{A}/l_{0}=0.01$; (**c**) $L_{A}/l_{0}=0.1$.

When discussing the maximum tuning effect of a tensile-mode MIAPB on the output current of the PN junction, it is enough to only investigate the situation occurring in the contact barrier region [35]. The $L_{A}/l_{0}$ values are 0, 0.01 or 0.1, and the changes in the carrier concentration distribution (corresponding to the maximum output current under conditions of varying barrier widths and barrier heights) are shown in Figure 2. The case where the loading center deviates from the interface is labeled as the offset state to simplify the analytical process. Carrier inversion occurs when the extent of mechanical loading reaches a certain level in a piezoelectric PN junction. Under these conditions, the majority carriers transform into minority carriers (and vice versa) in the local region [33]. From the degree of carrier inversion, it can be seen that—with the increase of the offset, when the maximum output current is reached—less and less majority carriers enter the contact barrier region and become unbalanced minority carriers. On the other hand, due to the influence of offset, the shielding effect of carriers on the left end of the MIAPB is weakened so that the barrier height required to reconstruct the contact barrier structure is reduced. For example, when $\Delta l=1.0l_{0}$ and the $L_{A}/l_{0}$ value increases from 0 to 0.1, the stress required to generate the maximum output current decreases from 17.19 MPa to 14.04 MPa.

**Figure 3:**
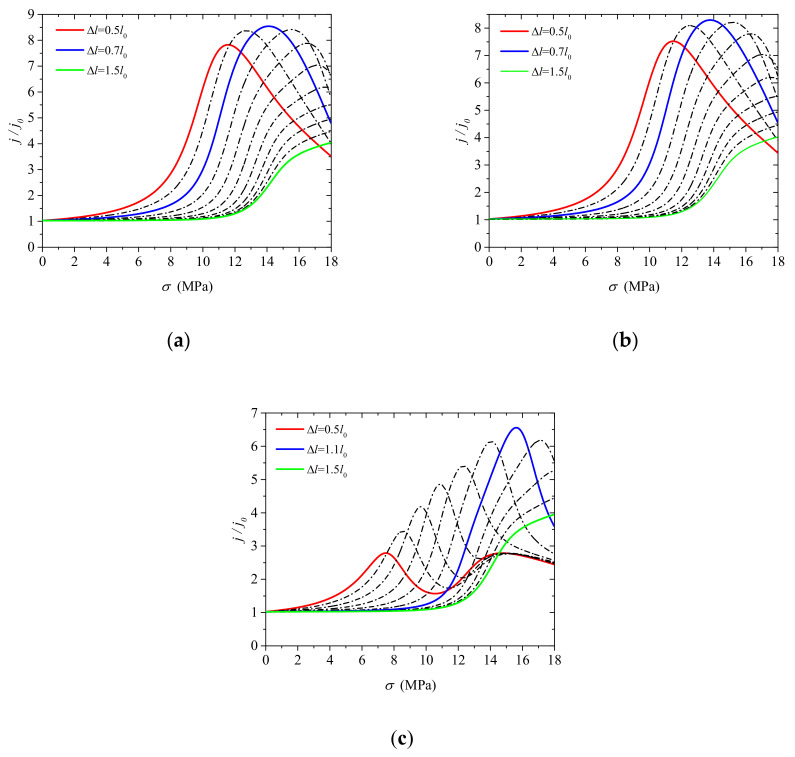
Variations in the output current density (at the PN junction) with stress under conditions of varying MIAPB positions. (**a**) $L_{A}/l_{0}=0$, (**b**) $L_{A}/l_{0}=0.01$, and (**c**) $L_{A}/l_{0}=0.1$.

The variation in the output current with stress under conditions of varying widths of MIAPB is shown in Figure 3. The output current initially increases and then decreases with an increase in stress. This can be attributed to the fact that the local majority and minority carriers in the PN junction are subjected to conditions of three different stages: concentration close (non-inversion), inversion and over-inversion. It should be noted that the carrier concentration near the interface is relatively low. Hence, the polarization electric field inside a narrow MIAPB cannot be sufficiently shielded. Under conditions of MIAPB asymmetry (on the two sides of the interface), consistent driving capabilities are not observed at the left and right ends. The carrier concentration at both ends of the MIAPB cannot reach the inversion state simultaneously under conditions of increasing loading. The greater the offset, the smaller the barrier width and the more prominent the effect (Figure 2b,c). It is observed that the enhancement effect exerted by the left and right ends of MIAPB on the output current of a PN junction is not synchronous. This significantly influences the *I*–*V* characteristics of the devices. The variations in the output current with the changes in the loading properties present a bimodal characteristic, as shown by the red curve in Figure 3c. To the best of our knowledge, we are the first to report this interesting phenomenon that can potentially affect the development of logic circuits or mechanical switches. It has also been observed that the reconstruction effect exerted on the barrier structure by an offset MIAPB is not as high as the effect exerted by a symmetrical one. As can be seen from the blue curves in Figure 3a–c, the maximum enhancement in the output current was recorded under conditions of symmetrical loading. The carrier distribution properties are presented in Figure 2, and the analysis of the figure reveals that the shielding effect exerted by carriers varies with the widths of the MIAPBs. The reconstruction effect exerted by a tensile-mode MIAPB varies with the width of the MIAPB. The larger the width of the contact barrier, the higher the degree of influence of the carrier shielding effect and the greater the required stress. The polarized electric field at both ends of MIAPB drives more carriers to participate in the process of redistribution. This reflects the difference in the effective mass of electrons and holes. It can be inferred that the shielding ability of the polarized electric field in the n-zone containing electrons is different than the shielding effect recorded for the p-zone with holes as the majority carriers. When the barrier width is large enough, a small degree of offset should be realized to achieve the maximum current gain effect [35]. However, the MIAPBs characterized by small barrier widths (e.g., $\Delta l=0.7l_{0}$) exert a strong regulation effect on the PN junction under conditions of low stress. Therefore, only the case where the MIAPB acts symmetrically on both sides of the interface is considered when the limiting gain effect (exerted by the tensile-mode MIAPB) on the output current of a PN junction is studied.

**Figure 4:**
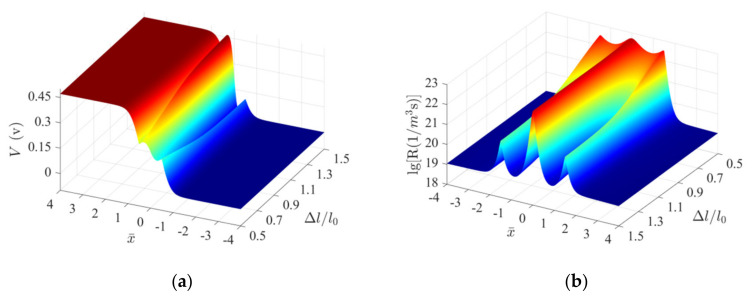
Evolution characteristics of the important physical quantities characterizing a ZnO PN junction subjected to a tensile-mode MIAPB under conditions of $L_{A}/l_{0}=0$. (**a**) Electric potential configurations and (**b**) recombination rates.

Figure 4 presents the distribution characteristics of electric potential and recombination rates. The curves were generated under conditions of a fixed action position ($L_{A}/l_{0}=0$) and fixed height (σ=9MPa) with varied barrier widths. The region $\bar{x}\leq 4$ is presented for clarity as insignificant changes in the physical quantities are observed beyond this region. The results presented in Figure 8 were obtained following the same processing method. As Δl is increased from $0.5l_{0}$ to $1.5l_{0}$, a pair of potential peaks and valleys form gradually at the interface ($L/l_{0}=0$), as shown in Figure 4a. When the MIAPB widens, the potential peaks and valleys gradually move away from the interface, and the reconfiguration effect exerted on the contact barrier is significantly weakened. A region of rapid recombination near the interface and two other regions characterized by high recombination rates are generated at the loading points as the width of MIAPB gradually increases (Figure 4b). The three-peak structure reflecting the distribution of the recombination rate became prominent, but the height of the peak reflecting the recombination rate decreased as the width of MIAPB increased. The reconstruction effect exerted on the contact barrier gradually decreases with an increase in the width of MIAPB. Under these conditions, the redistribution of charge carriers is promoted over a large range. The former and latter weaken and enhance the output current gain effect, respectively. It has been observed that these are competing mechanisms, which is the internal reason for the formation of the distribution trend of the above current-force curve. Therefore, the presence of an optimal barrier structure that helps generate the best tuning effect is confirmed.

**Figure 5:**
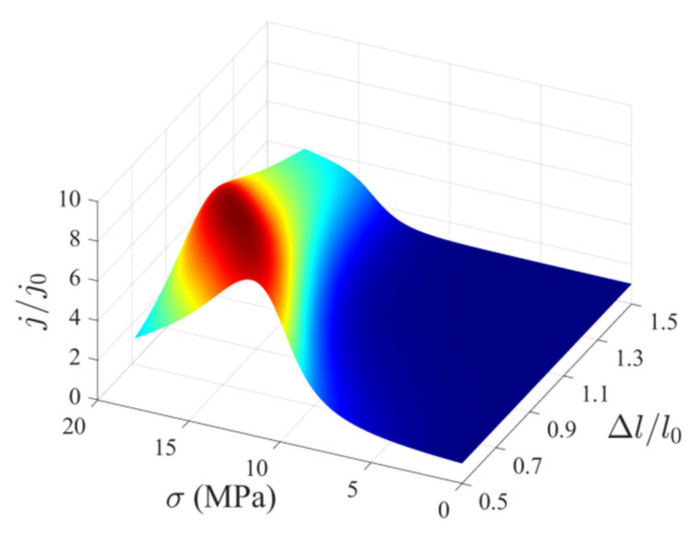
Variations in the output current at the PN junction with the variations in the height and width of tensile-mode MIAPB when $L_{A}/l_{0}=0$.

The variations in the output current of a PN junction with the variations in the width and height of MIAPB are presented in Figure 5 ($L_{A}/l_{0}=0$). The output current initially increases and then decreases with an increase in loading. These stages correspond to the non-inversion and over-inversion stages of the carriers near the loading points, respectively. The output current is analyzed, and it is observed that a wide MIAPB weakens the shielding effect of carriers, and the reconstruction of the contact barrier becomes increasingly difficult under these conditions. The stress required to reach the maximum output current gain increases, and the extent of gain realized decreases. Narrow MIAPBs exert a strong reconstruction effect on the PN junction. The barrier structure can be effectively adjusted when a small force is applied. The carriers are prone to over-inversion, and the strength of the enhancement effect exerted by MIAPB on the output current of the PN junction is weakened. In essence, the reconstruction of the contact barrier is also due to the redistribution of charge carriers. Namely, the range of carrier redistribution and over-inversion of local carriers forms a second competitive mechanism. Analysis of the two confinement mechanisms discussed above reveals an upper limit to the current gain achieved for the case of a tensile-mode MIAPB acting on PN junctions. The half-barrier width (Δl) and stress (σ) required for the generation of an optimal barrier structure were determined, and the values were calculated to be $0.71l_{0}$ and 14.25 MPa, respectively. The limiting output current (*j*/*j*_0_) was found to be 8.41.

### 3.3. Limit Tuning Effect Exerted by Compressive-Mode MIAPB

**Figure 6:**
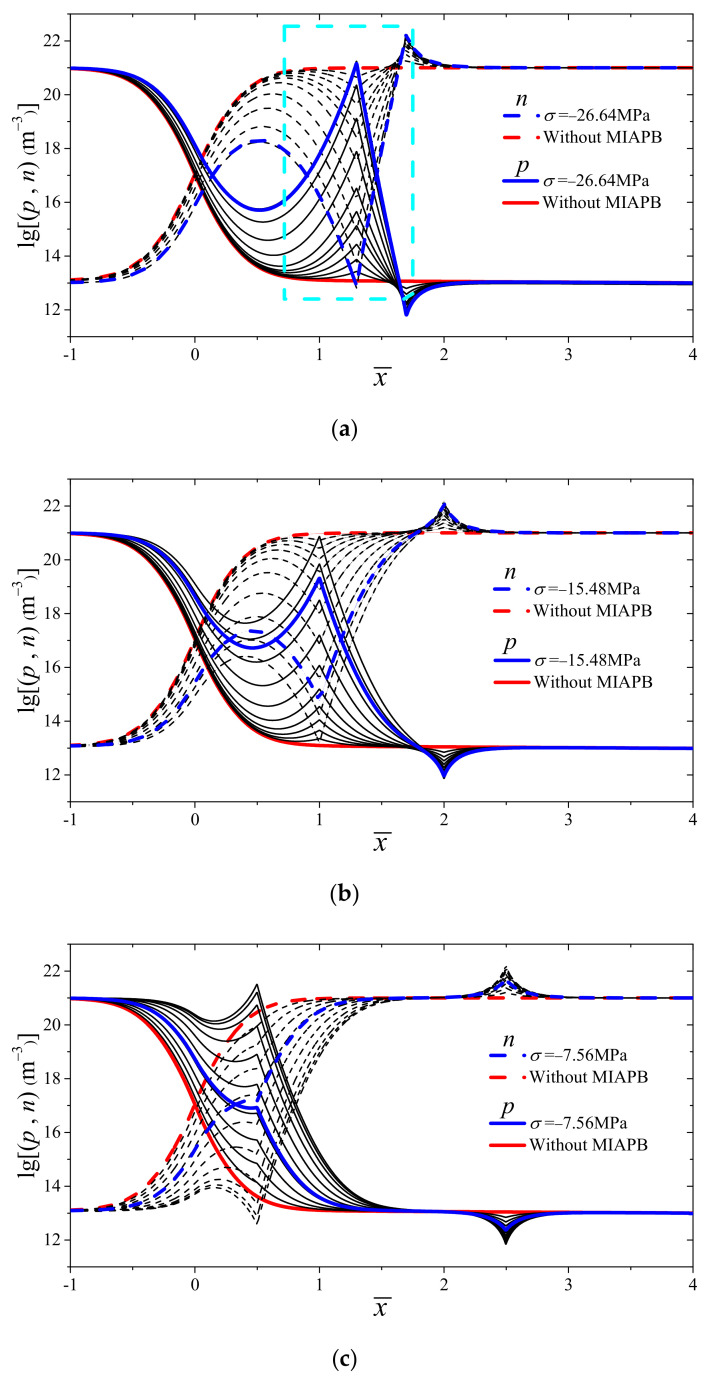
Changes in the carrier concentration when σ varies from 0 to 18 MPa (the left end of MIAPB is kept fixed, and the barrier width is varied). (**a**) $\Delta l=0.2l_{0}$; (**b**) $\Delta l=0.5l_{0}$; (**c**) $\Delta l=1.0l_{0}$.

The maximum degree of tuning effect is exerted on the *I*–*V* characteristics of the PN junction when the compressive-mode MIAPB acts outside the contact barrier region [35]. The changes in the carrier concentration with an increase in stress are shown in Figure 6. The changes are recorded for the case when MIAPB acts on the n-zone; the half-width of MIAPB (Δl) is equal to $0.2l_{0}$,$0.5l_{0}$ or $1.0l_{0}$; and the loading center is fixed at $L_{A}=1.5l_{0}$. The left and right ends of the system can interact with the contact barrier when the barrier width is small ($\Delta l=0.2l_{0}$). This can be attributed to the absence of enough carriers to shield the polarization electric field within the MIAPB. The left end of MIAPB promotes the transformation of majority carriers on both sides of the interface into minority carriers, while the right end generates a superposition effect (the superposition of MIAPB and the contact barrier increases the total barrier) with the contact barrier. With an increase in loading, the concentration difference between the majority and minority carriers decreases until inversion occurs. When the loading is increased further, a well of majority carriers is formed inside the barrier, as shown by the cyan square in Figure 6a. (A hole well is formed when MIAPB is located in the p-zone, and an electron well is formed when MIAPB is located in the n-zone.) This prevents the majority carriers from diffusing through the interface and transforming into minority carriers. It also results in the over-inversion of charge carriers. Under these conditions, the concentration difference between the majority and minority carriers is inversely increased. When the width of the MIAPB increases, the extent of the shielding effect exerted by carriers on the polarization electric field within the MIAPB gradually increases. It becomes increasingly difficult to form a well of the majority carriers under these conditions (Figure 6b,c). The sensitivity of the PN junction to the influence of MIAPB increases with an increase in the extent of interaction between the barriers. Unlike the case when $\Delta l=0.2l_{0}$ (or $0.5l_{0}$), a large number of carriers can pass through the interface as non-equilibrium minority carriers under the influence of a weak force when $\Delta l=1.0l_{0}$. The width of MIAPB increases, reducing the difference in the carrier concentration over a wider range.

**Figure 7:**
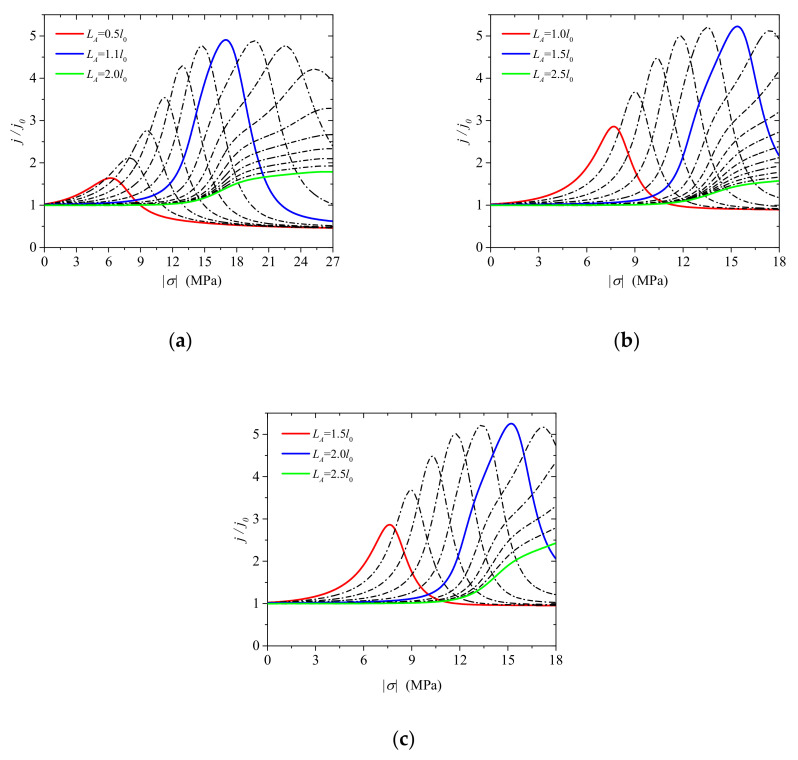
Variations in the output current density (at the PN junction) with stress under conditions of varying MIAPB widths. (**a**) $\Delta l/l_{0}=0.2$, (**b**) $\Delta l/l_{0}=0.5$ and (**c**) $\Delta l/l_{0}=1.0$.

The variation in the output current with stress is shown in Figure 7. Analysis of the figure reveals that the output current initially increases and then decreases with an increase in stress. The effect of MIAPB helps reduce the local concentration difference between the majority and minority carriers. It also helps improve the extent of recombination of carriers. The output current at the PN junction is improved under these conditions. The output current decreases with an increase in loading. This can be attributed to the over-inversion and superposition effects.

The influence of a narrow MIAPB ($\Delta l=0.2l_{0}$) presented near the interface helps in the production of majority carriers well, and the maximum output current generated at this point is significantly smaller than that generated under the action of a wider MIAPB ($\Delta l=0.5l_{0}$ or $1.0l_{0}$) (red curve; Figure 7a). Under conditions of large stress (σ>9MPa), the current even decreases below the initial current $j_{0}$. The maximum output current at the PN junction initially increases and then decreases as MIAPB moves away from the interface (Figure 7a–c). This can be attributed to the fact that the strength of the superposition effect exerted by MIAPB decreases when MIAPB moves away from the interface. The generation of the wells consisting of majority carriers becomes difficult under the influence of narrow MIAPB. Hence, the maximum output current associated with the PN junction increases in the first stage. When the MIAPB moves close to the boundary, the strength of interaction between the barrier and the contact barrier decreases significantly, and the maximum output current at the PN junction decreases. The shielding effect exerted by the carriers on the polarization electric field within the MIAPB increases, and the strength of the superposition effect decreases with an increase in the MIAPB width. The interaction between MIAPB and the contact barrier and the over-inversion of carriers predominantly influence the regulation effect of MIAPB when the MIAPB is significantly wide. The former and latter enhance and weaken the output current, respectively, and these are competing mechanisms. Since there is one less mechanism (majority carriers well are ignored at this point) that weakens the output current of the PN junction, the tuning effect of MIAPB is more significant with a larger ΔL. For example, when ΔL is equal to $0.2l_{0}$, $0.5l_{0}$ or $1.0l_{0}$, the stress required to reach the maximum output current is 26.64 MPa, 15.48 MPa and 7.56 MPa, respectively. The corresponding carrier distribution characteristics are shown in Figure 6 (blue curve).

Analysis of Figure 7a–c reveals that MIAPB should be close to the interface to exert a significant regulatory effect under conditions of a small ΔL. Therefore, the barrier width should be increased to improve the tuning effect. The carrier distribution range widens with an increase in the width of MIAPB. The larger the range, the lesser the concentration difference among carriers. This results in an increase in the area of the region characterized by a high recombination rate. The output current increases under these conditions. Analysis of the distribution of carriers (Figure 6) reveals that the region of over-inversion expands under these conditions, resulting in the weakening of the output current. These processes are competing mechanistic processes. The influence of MIAPB width on the output current of the PN junction is counteracted by the competing mechanism when the carriers effectively shield the polarization electric field present inside MIAPB. When $\Delta l=l_{0}$ the polarization electric field inside the MIAPB acting outside the space charge region is sufficiently shielded by carriers. The limit tuning effect exerted by MIAPB on the PN junction can be effectively studied under these conditions. Thus, two (the position and the height of the artificial potential barrier) and not three factors affect the tuning effect exerted by MIAPB on the *I*–*V* characteristics of the PN junction.

**Figure 8:**
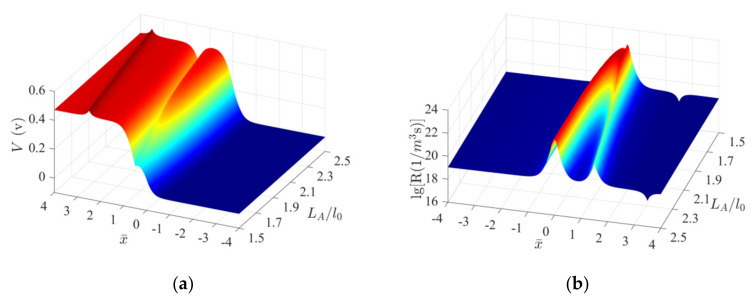
Evolution characteristics of the important physical quantities characterizing a ZnO PN junction subjected to a compressive-mode MIAPB under conditions of $\Delta l=1.0l_{0}$. (**a**) Electric potential configurations and (**b**) recombination rates.

Figure 8 presents the changes in the electric potential and recombination rates when a fixed-width ($\Delta l=l_{0}$) MIAPB moves over the PN junction ($\left|\sigma \right|=9\mathrm{MPa}$). Only the case where loading acts on the n-zone is considered, as similar observations are made for the p-zone. A potential peak and a potential valley gradually form at the interface ($L/l_{0}=0$) when $L_{A}$ increases from $1.5l_{0}$ to $2.5l_{0}$ (Figure 8a).

The increase in the potential at the potential peak results in a decrease in the potential energy of the electrons. The electron concentration decreases under these conditions, reducing the concentration difference between the majority (electrons) and minority carriers (holes). This eventually results in an increase in the carrier recombination rate and the output current at the PN junction. An opposite phenomenon occurs at the potential valley. The progress of the two competing mechanisms results in the generation of an optimal barrier structure when the compressive-mode MIAPB interacts with the contact barrier. Figure 8b presents the influence of the competing mechanisms and affects the trend of the above current-force curves. Under these conditions, a peak of progressively decreasing intensity is generated. The double-peak structure of the recombination rate explains why the compressive-mode MIAPB can effectively tune the output current at the PN junction when it functions outside the contact barrier region.

**Figure 9:**
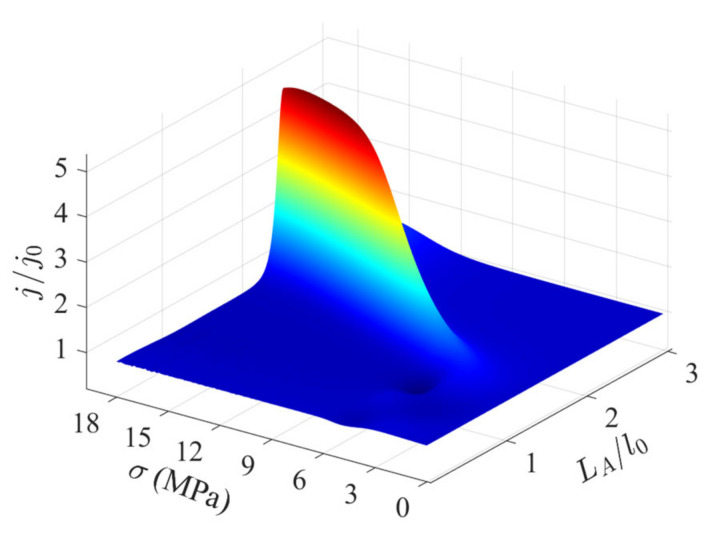
Variations in the output current at the PN junction with the variations in the height and position of the compressive-mode MIAPB when $\Delta l=1.0l_{0}$.

Figure 9 presents output current as a function of the action position and barrier height of MIAPB ($\Delta l=l_{0}$). The strong interaction between MIAPB and contact barrier leads to the enhancement of the current; the superposition effect leads to the weakening of the current; and the two affected by the position and height of the artificial barrier contribute to the second competing mechanism. The output current of the PN junction will have an extreme value as the barrier height of MIAPB increases under suitable barrier position. It has been numerically verified that the maximum output current ($j/j_{0}=5.18$) is generated at the PN junction when $L_{A}=1.99l_{0},\;\sigma =15.03\mathrm{MPa}$.

## 4. Conclusions

The formation of MIAPB is induced when mechanical loadings act on a piezoelectric PN junction. The significant changes in the properties of related physical quantities at the interface were studied. It was observed that three factors (barrier width, height and position of MIAPB) affected the tuning effect exerted by MIAPB on the *I*–*V* characteristics of PN junctions. We conducted an in-depth analysis of these factors and presented the results:

1. The width, height and position of the MIAPB affect the extent of interaction between the MIAPB and the contact barrier. Different interaction intensities, superposition effects, carrier inversion degrees and carrier redistribution ranges are realized. These factors, coupled with each other, result in the generation of the current gain effect of the PN junction. Multiple competition mechanisms are observed, forming the physical basis of the limit tuning effect exerted by MIAPB on the *I*–*V* characteristics of the PN junction.
2. Output current will appear in novel bimodal curves in the process of force regulation under small offset state tensile-mode MIAPB and even below the initial current with action of a narrow compressive-mode MIAPB. These phenomena have potential application value in logic circuits and mechanical switches.
3. The inconsistency between the tensile-mode MIAPB under the offset state and the contact barrier confines the tuning effect exerted by MIAPB on the output current of a PN junction. The optimal loading method involves the symmetrical introduction of the tensile-mode MIAPB on both sides of the interface. As for compressive-mode MIAPB, the generation of the optimal barrier can be attributed to the carrier redistribution range and the carrier inversion degree. Therefore, the best tuning effect is generated under conditions of an optimal barrier width range.

## Data Availability

Not applicable.

## References

[1] Lee K.Y., Kumar B., Seo J.S., Kim K.-H., Sohn J.I., Cha S.N., Choi D., Wang Z.L., Kim S.-W. (2012). P-type polymer-hybridized high-performance piezoelectric nanogenerators. Nano Lett..

[2] Gao Y., Wang Z.L. (2007). Electrostatic potential in a bent piezoelectric nanowire. The fundamental theory of nanogenerator and nanopiezotronics. Nano Lett..

[3] Falconi C. (2019). Piezoelectric nanotransducers. Nano Energy.

[4] Koka A., Sodano H.A. (2013). High-sensitivity accelerometer composed of ultra-long vertically aligned barium titanate nanowire array. Nat. Commun..

[5] Zhu P., Zhao Z.M., Nie J.H., Hu G., Li L., Zhang Y. (2018). Ultra-high sensitivity strain sensor based on piezotronic bipolar transistor. Nano Energy.

[6] Hossain K.M., Ahmed H.M., Khan I.M., Miah M.S., Hossain S. (2021). Recent Progress of Rare Earth Oxides for Sensor, Detector, and Electronic Device Applications: A Review. ACS Appl. Electron. Mater..

[7] Xu C.Y., Wei P.J., Wei Z.B., Guo X. (2022). Shear horizontal wave in a piezoelectric semiconductor substrate covered with a metal layer with consideration of Schottky junction effects. Appl. Math. Model..

[8] White D.L. (1962). Amplification of ultrasonic waves in piezoelectric semiconductors. J. Appl. Phys..

[9] Hutson A.R., White D.L. (1962). Elastic wave propagation in piezoelectric semiconductors. J. Appl. Phys..

[10] Xiao G., Wei P.J. (2021). Dispersion relations of in-plane elastic waves in nano-scale one dimensional piezoelectric semiconductor/piezoelectric dielectric phononic crystal with the consideration of interface effect. Appl. Math. Model..

[11] Schülein F.J.R., Müller K., Bichler M., Koblmüller G., Finley J.J., Wixforth A., Krenner H.J. (2013). Acoustically regulated carrier injection into a single optically active quantum dot. Phys. Rev. B.

[12] Büyükköse S., Hernández-Mínguez A., Vratzov B., Somaschini C., Geelhaar L., Riechert H., van der Wiel W.G., Santos P.V. (2014). High-frequency acoustic charge transport in GaAs nanowires. Nanotechnology.

[13] Li L.H., Hao M.M., Yang X.Q., Sun F., Bai Y., Ding H., Wang S., Zhang T. (2020). Sustainable and flexible hydrovoltaic power generator for wearable sensing electronics. Nano Energy.

[14] Wang Z.L. (2014). Piezotronics and Piezo-Phototronics.

[15] Zhang Y., Liu Y., Wang Z.L. (2011). Fundamental theory of piezotronics. Adv. Mater..

[16] Zhao L.N., Wei P.J., Huang M.S., Xu Y.Q. (2022). Electro-Thermo-Mechanical multiple fields coupled wave propagation through piezoelectric semiconductor sandwich structure. Compos. Struct..

[17] Cheng R.R., Zhang C.L., Chen W.Q., Yang J. (2019). Electrical behaviors of a piezoelectric semiconductor fiber under a local temperature change. Nano Energy.

[18] Yang Z., Sun L., Zhang C.L., Gao C. (2022). Analysis of a composite piezoelectric semiconductor cylindrical shell under the thermal loading. Mech. Mater..

[19] Liang C., Zhang C.L., Chen W.Q., Yang J. (2020). Electrical response of a multiferroic composite semiconductor fiber under a local magnetic field. Acta Mech. Solida Sin..

[20] Mortazavi B., Shojaei F., Yagmurcukardes M., Shapeev A.V., Zhuang X. (2022). Anisotropic and outstanding mechanical, thermal conduction, optical, and piezoelectric responses in a novel semiconducting BCN monolayer confirmed by first-principles and machine learning. Carbon.

[21] Hossain K.M., Raihan A.G., Akbar A.M., Rubel M.H.K., Ahmed M.H., Khan M.I., Hossain S., Sen S.K., Jalal M.I.E., El-Denglawey A. (2022). Current Applications and Future Potential of Rare Earth Oxides in Sustainable Nuclear, Radiation, and Energy Devices: A Review. ACS Appl. Electron. Mater..

[22] Wang J.Q., Wang H.Y., Li X.Y., Zi Y. (2019). Self-powered electrowetting optical switch driven by a triboelectric nanogenerator for wireless sensing. Nano Energy.

[23] Lee K.Y., Bae J., Kim S.M., Lee J.-H., Yoon G.C., Gupta M.K., Kim S., Kim H., Park J., Kim S.-W. (2014). Depletion width engineering via surface modification for high performance semiconducting piezoelectric nanogenerators. Nano Energy.

[24] Shin Y.H., Choi J., Kim S.J., Kim S., Maurya D., Sung T.H., Priya S., Kang C.Y., Song H.C. (2020). Automatic resonance tuning mechanism for ultra-wide bandwidth mechanical energy harvesting. Nano Energy.

[25] Qu Y.L., Jin F., Yang J.S. (2022). Torsion of a piezoelectric semiconductor rod of cubic crystals with consideration of warping and in-plane shear of its rectangular cross section. Mech. Mater..

[26] Salah B.I., Takali F., Othmani C., Njeh A. (2022). SH waves in a stressed piezoelectric semiconductor plates: Electron and hole drift phenomenon. Int. J. Mech. Sci..

[27] Liang Y.X., Hu Y.T. (2020). Effect of interaction among the three time scales on the propagation characteristics of coupled waves in a piezoelectric semiconductor rod. Nano Energy.

[28] Huang H.Y., Qian Z.H., Yang J.S. (2019). *I-V* characteristics of a piezoelectric semiconductor nanofiber under local tensile/compressive stress. J. Appl. Phys..

[29] Fan S.Q., Hu Y.T., Yang J.S. (2019). Stress-induced potential barriers and charge distributions in a piezoelectric semiconductor nanofiber. Appl. Math. Mech..

[30] Huang K., Han R.Q. (2015). The Physical Basis of Semiconductors.

[31] Fan S.Q., Yang W.L., Hu Y.T. (2018). Adjustment and control on the fundamental characteristics of a piezoelectric PN junction by mechanical-loading. Nano Energy.

[32] Yang W.L., Liu J.X., Xu Y.L., Hu Y.T. (2020). A full-coupling model of PN junctions based on the global-domain carrier motions with inclusion of the two metal/semiconductor contacts at endpoints. Appl. Math. Mech..

[33] Yang W.L., Liu J.X., Hu Y.T. (2021). Mechanical tuning methodology on the barrier configuration near a piezoelectric PN interface and the regulation mechanism on *I−V* characteristics of the junction. Nano Energy.

[34] Yang G.Y., Yang L., Du J.K., Wang J., Yang J. (2020). PN junctions with coupling to bending deformation in composite piezoelectric semiconductor fibers. Int. J. Mech. Sci..

[35] Yang Y.Z., Yang W.L., Wang Y.B., Zeng X., Hu Y. (2022). A mechanically induced artificial potential barrier and its tuning mechanism on performance of piezoelectric PN junctions. Nano Energy.

[36] Cheng R.R., Zhang C.L., Chen W.Q., Yang J. (2020). Temperature effects on PN junctions in piezoelectric semiconductor fibers with thermoelastic and pyroelectric couplings. J. Electron. Mater..

[37] Guo M.K., Lu C.S., Qin G.S., Zhao M. (2021). Temperature gradient-dominated electrical behaviours in a piezoelectric PN junction. J. Electron. Mater..

